# Comment on “Ethical and legal considerations of the use of robotic surgery in Brazil”

**DOI:** 10.1590/0100-6991e-20243855-en

**Published:** 2024-12-02

**Authors:** ANTONIO FERREIRA COUTO

**Affiliations:** 1 - Colégio Brasileiro de Cirurgiões, Departamento Jurídico - Rio de Janeiro - RJ - Brasil

Firstly, it is commendable that the CBC has taken the initiative to issue the Robotic Surgery Certification Guidelines^1^. As early as 2020, we were already considering this topic with the understanding that it represented a significant advancement. Indeed, Common Law will consistently follow societal developments and precede statutory law. The well-crafted article^2^ provides an insightful exploration of Civil Liability, particularly when highlighting the assumption of new risks and the imperative to understand the ethical and legal implications associated with this practice.

Robotic surgeries present unique challenges that impact the assessment and determination of responsibilities. Traditionally, the team leader is held primarily responsible for all stages of the procedure, including those performed by the team. In robotic surgery, it is important to highlight to the judiciary that the robotic surgeon, or console surgeon, maintains part of their face in contact with the machine’s display throughout the surgical process. The robot ceases its actions when this contact is interrupted. This scenario alters the supervision dynamics not only over the surgical team but also draws attention away from perioperative events. Therefore, it is essential to consider this factor when evaluating the existence of a causal link concerning the surgeon’s liability. This new element introduced by the machine warrants further study and the development of new doctrines.

Another significant aspect involves the operating room nurse, a professional qualified by the manufacturer to manage the external movements of the robot. This creates a new and important focus of judicial observation in determining responsibilities. Since the surgeon does not participate in the docking process, any failures in the machine’s use fall under the nurse’s responsibility. The nurse is accountable for these actions both as an agent before the civil court and in cases where conduct may constitute criminal offense.

The Technical Director of the high-complexity hospital where the robotic surgery will be conducted bears the responsibilities as outlined in Article 5 and its sole paragraph of Resolution CFM-2311/2022, dated 03/28/2022. It should be noted that, under these circumstances, the surgeon will not be held liable for any procedural failures.

The anesthetic protocols in robotic surgeries involve variations in drug dosages, which are often unfamiliar to legal professionals.

Expectations concerning the use of sensitive patient data by the manufacturer should not be attributed to the surgeon.

A notable point to consider is the presence of an electronic engineer in the operating room. This individual, employed by the manufacturer, is responsible for managing both hardware and software. According to Articles 932 and 933 of the Brazilian Civil Code, this responsibility lies solely with the electronic engineer and does not extend to the surgeon.

Emphasizing the vast and uncharted realm of robotics and its implications on Brazilian Civil Liability, the CBC’s Legal Department stands ready to provide guidance and support to its members. We aim to address the uncertainties that arise as they continually strive for cures and advancements.
